# Proteomic Interrogation of Androgen Action in Prostate Cancer Cells Reveals Roles of Aminoacyl tRNA Synthetases

**DOI:** 10.1371/journal.pone.0007075

**Published:** 2009-09-18

**Authors:** Adaikkalam Vellaichamy, Arun Sreekumar, John R. Strahler, Theckelnaycke Rajendiran, Jindan Yu, Sooryanarayana Varambally, Yong Li, Gilbert S. Omenn, Arul M. Chinnaiyan, Alexey I. Nesvizhskii

**Affiliations:** 1 Michigan Center for Translational Pathology, University of Michigan, Ann Arbor, Michigan, United States of America; 2 Department of Pathology, University of Michigan, Ann Arbor, Michigan, United States of America; 3 Center for Computational Medicine and Bioinformatics, University of Michigan, Ann Arbor, Michigan, United States of America; 4 Internal Medicine and Human Genetics, University of Michigan, Ann Arbor, Michigan, United States of America; 5 Michigan Proteome Consortium, University of Michigan, Ann Arbor, Michigan, United States of America; 6 Comprehensive Cancer Center, University of Michigan, Ann Arbor, Michigan, United States of America; 7 Northwestern University Feinberg School of Medicine, Chicago, Illinois, United States of America; Yale University, United States of America

## Abstract

Prostate cancer remains the most common malignancy among men in United States, and there is no remedy currently available for the advanced stage hormone-refractory cancer. This is partly due to the incomplete understanding of androgen-regulated proteins and their encoded functions. Whole-cell proteomes of androgen-starved and androgen-treated LNCaP cells were analyzed by semi-quantitative MudPIT ESI- ion trap MS/MS and quantitative iTRAQ MALDI- TOF MS/MS platforms, with identification of more than 1300 high-confidence proteins. An enrichment-based pathway mapping of the androgen-regulated proteomic data sets revealed a significant dysregulation of aminoacyl tRNA synthetases, indicating an increase in protein biosynthesis- a hallmark during prostate cancer progression. This observation is supported by immunoblot and transcript data from LNCaP cells, and prostate cancer tissue. Thus, data derived from multiple proteomics platforms and transcript data coupled with informatics analysis provides a deeper insight into the functional consequences of androgen action in prostate cancer.

## Introduction

Cancer of the prostate (PCa) is the most commonly diagnosed cancer among men in the United States, with an estimated 200,000 new cases and 29,000 deaths for 2008 [1]. Many reports suggest that androgens which are required for normal development of the prostate also support the growth and progression of PCa. Androgens exert their cellular effects via binding to the androgen receptor (AR), a member of the nuclear hormone receptor (NR) super family. AR, in turn, binds to androgen response elements (AREs) in the promoter and enhancer regions of target genes, acting as a transcriptional regulator by transducing the cognate hormone signal in the nucleus [2], [3]. Prostate cancer therapy through androgen ablation initially achieves regression of tumors; however, a more aggressive, hormone-refractory form of the tumor (HR-PCa) may evolve, with subsequent mortality. Although multiple groups have interrogated androgen-regulated changes at the transcriptome and proteome levels using gene expression arrays and mass spectrometry, respectively [4], [5], [6], [7], [8], [9], [10], there is still a lack of considerable understanding on the functional consequence of the hormone action during prostate cancer development. Partly this deficiency is a reflection of the sparseness in the proteomic data; due to the limitations of proteomic technologies, the total number of proteins identified and quantified in a single study is modest. In addition, there are technical challenges from such under-representation of proteins and in assessing the statistical significance of differences in protein expression.

To overcome these challenges, and to obtain a deeper insight into androgen-regulated proteomic alterations in prostate cancer, we applied a two-pronged strategy. First, we enhanced the breath of the androgen-regulated proteome profiled in this study by employing two complementary mass-spectrometry (MS) platforms: one that involves isotope labeling of peptides with an iTRAQ reagent followed by 2D liquid chromatography (LC) MALDI- TOF MS/MS analysis on an ABI 4800 MALDI tandem time-of flight (TOF/TOF) instrument [11] and the other a label-free approach based on spectral counting [12], [13], [14], [15] using Multi-dimensional Protein Identification Technology (MudPIT) [16] with Electrospray Ionization (ESI)- ion trap MS/MS on a linear ion trap (LTQ) instrument. Second, we elucidated the proteomic alterations in the context of functional pathways using Molecular Concepts Mapping (MCM, also called Oncomine Concepts Mapping (OCM)) [17], to gain deeper insight into the biology of androgen action in prostate cancer. Notably, such combinatorial analyses revealed a striking increase in the level of aminoacyl tRNA synthetases (aaRS) that could underlie the increase in protein biosynthesis predicted in prostate cancer by various gene expression studies.

## Results

**Figure 1:**
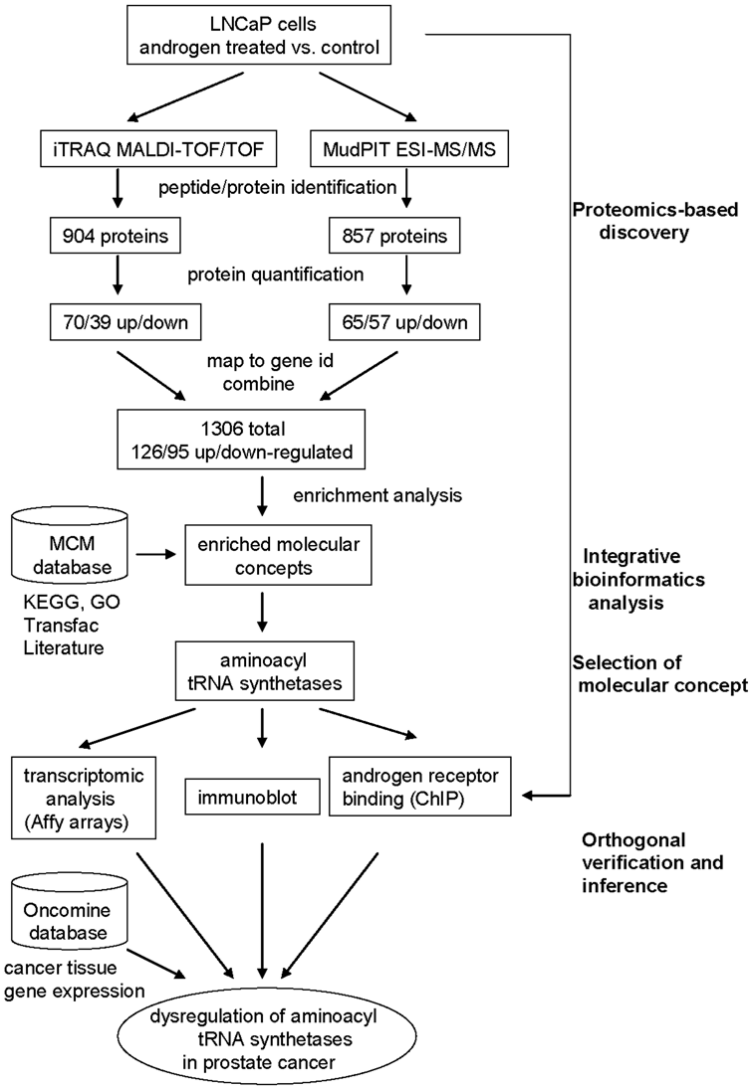
Outline of the strategy employed in this study (see text for details). Androgen-stimulated LNCaP cells were profiled using both quantitative iTRAQ MALDI- TOF/TOF and semi-quantitative MudPIT ESI- LTQ proteomics platforms. Data from each of these platforms were normalized independently and combined to generate a list of androgen-regulated proteins. This list was interrogated for biological associations using Molecular Concepts Mapping (MCM). The concept describing aminoacyl tRNA synthetases was selected for further examination; selected proteins were validated using immunoblot and immunofluorescence staining. Transcriptomic profiling and chromatin immunoprecipitation showed that androgen drives expression of aminoacyl-tRNA synthases at the transcript level. This was further confirmed using cancer tissue gene expression data and immunoblot analysis on prostate tissue samples, which demonstrated the existence of the elevated aminoacyl-tRNA synthase niche during prostate cancer progression.

We employed an integrative strategy (Figure 1) to understand androgen action in prostate cancer. In brief, we performed mass spectrometry-based proteome profiling of androgen-deprived and androgen-stimulated LNCaP cells. Trypsin-digested proteins were identified and quantified using two mass spectrometric platforms: iTRAQ MALDI-TOF MS/MS and MudPIT ESI- ion trap MS/MS. The data from each of these platforms were normalized independently and combined to generate a list of androgen regulated proteins. The androgen regulated proteome was interrogated for biological associations using Molecular Concepts Mapping (MCM). From the many molecular concepts, including the androgen-regulated and prostate cancer concepts that were enriched by our androgen up-regulated data set, the concept describing elevated aminoacyl tRNA synthetases (aaRSs) was selected for further examination. A subset of these proteins was validated by immunoblot analysis. Using transcriptomic profiling and chromatin immunoprecipitation, we showed that androgen drives the expression of aaRSs at the transcript level. Finally, we showed the existence of the elevated aaRS niche during prostate cancer progression using clinical specimens.

### Identification and relative quantitation of LNCaP proteins by iTRAQ MALDI-TOF MS/MS

**Figure 2:**
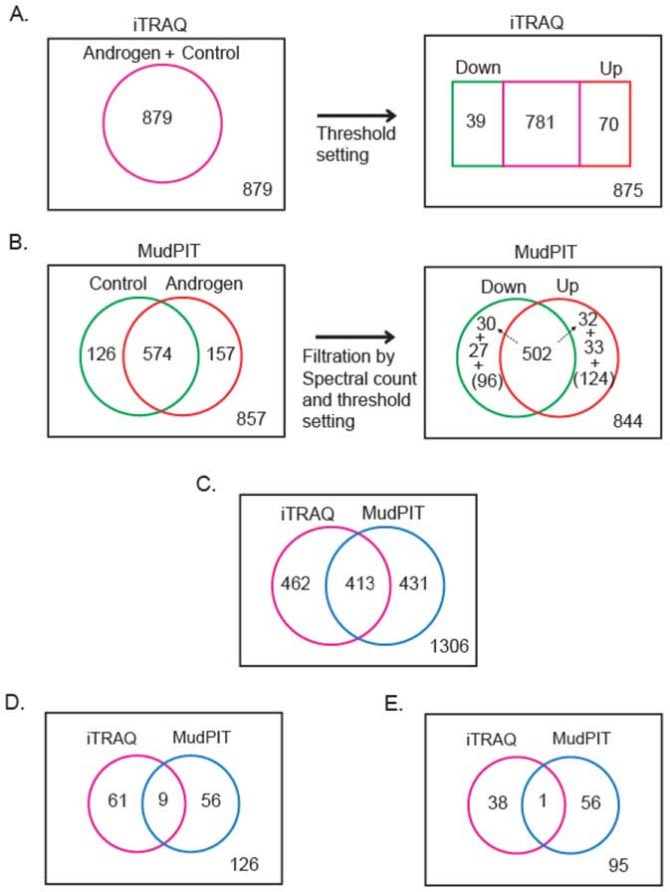
Total and androgen-regulated proteins identified from two mass-spectrometric platforms. A) Venn diagram showing the number of total and the sub-sets of androgen-regulated proteins identified from iTRAQ MALDI TOF- MS/MS analysis. A minimum threshold of 1.25 SD from the global mean in both androgen-treated samples and not less than 1.5 SD in one of the androgen treated samples was set. B) Venn diagram showing the numbers of total and androgen-regulated LNCaP proteins identified in MudPIT (ESI ion trap- MS/MS) analysis. Proteins shown in brackets were not considered for differential analysis because of their lower spectral counts. C) Venn diagram showing the total number of proteins identified from two mass-spectrometric platforms and their overlap. D) Venn diagram showing the total number of androgen up-regulated proteins identified from two mass-spectrometric platforms and their overlap. E. Venn diagram showing the total number of androgen down-regulated proteins identified from two mass-spectrometric platforms and their overlap.

Using iTRAQ MALDI-TOF MS/MS platform, we identified 904 proteins with high confidence scores as determined with SEQUEST [18], PeptideProphet [19] and ProteinProphet [20] computational tools. Of these, 879 were quantified post-data normalization (Figure 2A), with the overall protein identification FDR of less than 1 % (see Materials and Methods for detail).

Procedurally, the androgen proteome was assessed by iTRAQ using two independent biological replicates. A double duplex iTRAQ experiment was carried out wherein androgen-treated samples in each replicate were labeled with the 115 and 117 isobaric tags while their corresponding control samples were labeled using the 114 and 116 isobaric tags. In order to derive a threshold to define protein expression changes, we normalized the relative protein ratio for each of the androgen-treated samples and one of the controls (labeled with isobaric tag 116) to the protein expression in the control sample labeled with the 114 isobaric tag. The distribution of all protein ratios for the androgen-treated sample (tag 115) versus control (tag 116) is shown in Supplementary Figure S1A. More than 80% of proteins had ratios within the range of 0.8–1.2. The median-centered normalization resulted in a mean close to unity for all samples, and the standard deviation of the control replicate (tag 116, SD = 0.16) was used as a scale to set the threshold ratio for differentially expressed proteins. Accordingly, changes with a minimum threshold of 1.25 SD (ratio of 1.2 for up-regulated and 0.83 for down-regulated) were considered as significant (see Materials and Methods for details). The lists of all 70 androgen up-regulated and 39 down-regulated proteins identified from this analysis are given in Supplementary Table S1 and Supplementary Table S2, respectively.

### Semi-quantitative comparison of proteins using MudPIT analysis

Using MudPIT ESI- ion trap MS/MS proteomic profiling approach 857 proteins were identified with the minimum protein probability score of 0.9 (estimated FDR of less than 1%); 67% (574) of them were identified in common between control and androgen-treated samples, while 126 proteins and 157 proteins were identified only in control and androgen-treated samples, respectively (Figure 2B). In addition to determining the presence or absence of proteins, we performed a statistical analysis to compare the relative spectral counts for proteins in each treatment. The total number of spectra from either control or androgen-treated data was independently normalized, and the normalized cumulative spectral counts of all the peptides for each protein were used as a measure of protein abundance. Based on the statistical analysis of data, proteins were designated as differentially expressed if they were identified only in either the control (down-regulated) or androgen treated sample (up-regulated) with four or more peptides. For proteins identified in both samples, normalized spectral count ratios of two standard deviations above or below the mean of all proteins in that group (which corresponds to a four-fold change) was used as cut-off (see Materials and Methods; see Supplementary Figure S1B for distribution of the normalized spectral count ratios for proteins identified in control and androgen-treated samples). Based on these criteria, 65 and 57 proteins were designated as androgen up-regulated and down-regulated, respectively (Supplementary Table S3, upplementary Table S4).

### Combined list of differentially expressed proteins identified from the two MS platforms

With the understanding that the list of peptides profiled by mass spectrometry is influenced by the ionization source and the mass analyzer [21] (in this case MALDI- TOF vs. ESI-ion trap), we combined the protein lists derived from the two MS platforms to derive a composite list. This was facilitated by the fact that the two platforms identified similar numbers of high confidence proteins (857 and 879 proteins from MudPIT and iTRAQ, respectively, with comparable FDR of less than 1 %). As a first step in the process, non-redundant proteins were culled from each experiment by converting their IPI accession numbers to gene IDs. Three proteins (lectin, mannose-binding 2, LMAN2; CLPX, caseinolytic peptidase X homolog; and 40S ribosomal protein S9, RPS9) that showed discordant trends in their expression between the two platforms were removed. These procedures yielded a total of 844 and 875 proteins, respectively, for MudPIT and iTRAQ data sets (Figure 2B, 2A). Together 1306 proteins were observed in the combined dataset (Figure 1 and Figure 2C). Of these, 126 unique proteins were elevated upon androgen treatment. Notably, 9 of these androgen-up regulated proteins overlapped between the two proteomic platforms (Figure 2D): KLK3, ACSL3, FASN, FKBP5, AARS (alanyl-tRNA synthetase), FDFT1 (farnesyl-diphosphate farnesyltransferase 1), UAP1 (UDP-N-acteylglucosamine pyrophosphorylase 1), ENDOD1 (endonuclease domain containing 1), and NDRG1. The combined down-regulated list contained 95 proteins, and one (HNRPL, heterogeneous nuclear ribonucleoprotein L isoform a) was common between iTRAQ and MudPIT platforms (Figure 2E).

**Figure 3:**
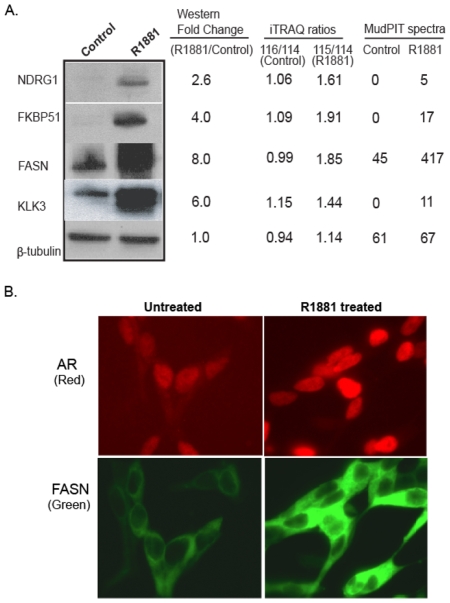
Androgen-induced expression of LNCaP proteins. A) Expression fold change of androgen-regulated proteins in mass-spectrometric quantitation compared to immunoblot assessment. Shown are the immunoblot bands and their intensities for androgen up-regulated proteins and their calculated intensity ratios as well as iTRAQ ratios and MudPIT spectral counts for the corresponding proteins. Beta-tubulin is used as a sample loading control. B) Immunofluorescence staining of AR (Red) and FASN (Green) following vehicle (ethanol) and 1 nM R1881 treatments for 48 h on LNCaP cells. Treatment was given after 48 h of androgen deprivation.

Our list of up-regulated proteins included five proteins that have been shown previously to be elevated by androgen action: ACSL3 (iTRAQ ratio 2.76), FKBP51 (iTRAQ ratio 1.91; MudPIT spectral counts 17∶0), FASN (iTRAQ ratio 1.85; MudPIT spectral counts 417∶45), NDRG1 (iTRAQ ratio 1.76; MudPIT spectral counts 5∶0), and KLK3 (also known as PSA, Prostate Specific Antigen[22], iTRAQ ratio 1.44; MudPIT ratio 11∶0). A direct quantitative comparison of proteins identified on both the MS platforms showed a high positive correlation (Rˆ2 = 0.92) for three proteins (ACSL3, FASN, and AARS; see Supplementary figure S1). These findings independently validated our normalization procedure and the thresholds that were used to build the combined list of androgen-regulated proteins. Further, immunoblot analysis was employed to confirm the androgen-regulation of a subset of up-regulated proteins (Figure 3A). Notably, in addition to validating our mass spectrometry results, the immunoblot analysis revealed significant compression in the fold change of protein expression delineated by iTRAQ experiment (Figure 3A). A similar orthogonal verification performed for the androgen up-regulated protein fatty acid synthase by immunofluorescence- microscopy is shown in Figure 3B.

### Analysis of androgen up-regulated biological processes and pathways by MCM analysis

**Figure 4:**
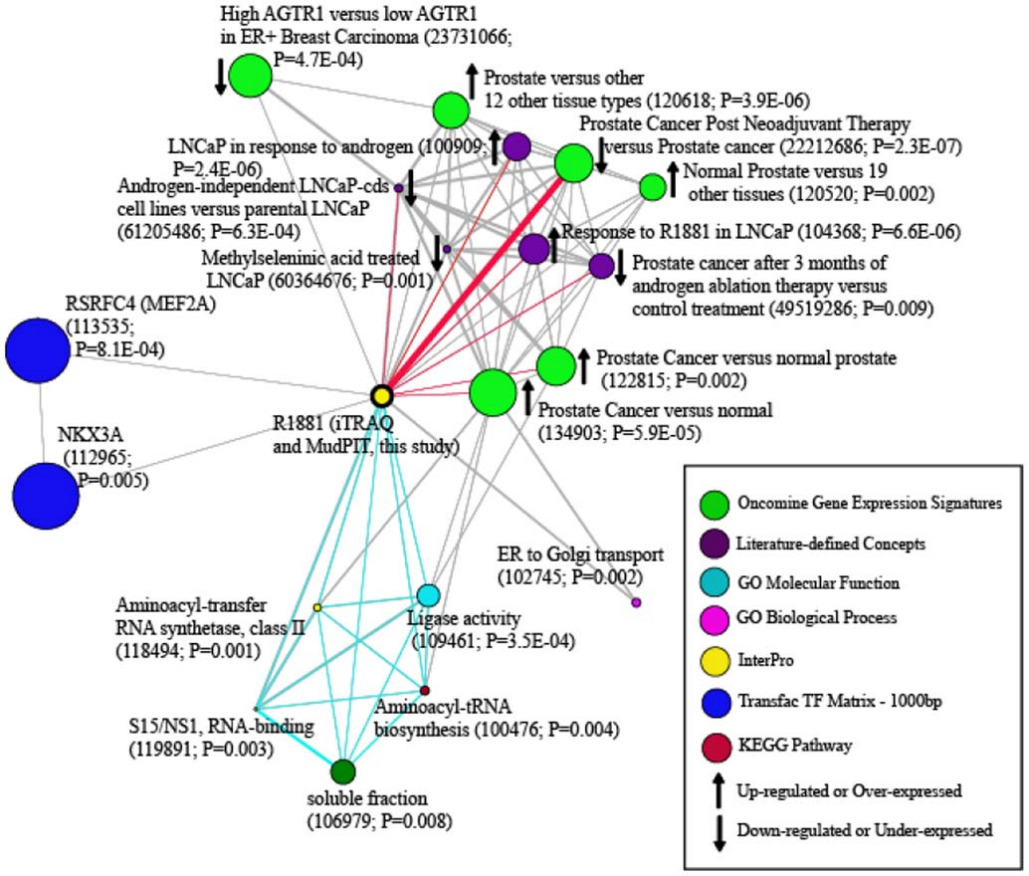
Molecular concepts of androgen up-regulated proteins from two mass spectrometry platforms. Network view of molecular concepts or sets of biologically related genes enriched in androgen up-regulated protein set obtained through Molecular Concepts Mapping (MCM) analysis. Each node represents a biological concept; the node size is proportional to the number of genes in each concept. Each edge represents a statistically significant enrichment. P-value of each concept and the MCM number of the concept are given in brackets. The most enriched concept is indicated by a thick edge. Red colored edges indicate enrichment of known prostate cancer-specific and androgen-regulated concepts. Interconnected aminoacyl tRNA synthetase concepts are indicated in blue edges at the bottom of the network.

To gain insight into the functional consequence of androgen-action, A large-scale association analysis was performed comparing our androgen up-regulated protein data set of 126 proteins with each of the 13364 concepts in the MCM [23]. The procedure involved in selection of significant molecular concepts is described in ‘Materials and Methods’ section. The enrichment network resulted in the MCM analysis with the combined set of androgen up-regulated proteins is presented in Figure 4. Importantly, the analysis showed enrichment of known prostate cancer-specific and androgen-regulated concepts, thus providing a validation of the proteomic data generated in this work (Figure 4, red colored edges). Nested within these previously known prostate-specific concepts was one that described a set of co-enriched genes down-regulated in prostate cancer patients after post-neoadjuvant (anti-androgen) therapy [17]. Additional concepts of significance included those for aminoacyl-tRNA biosynthesis and ER to Golgi transport (both GO biological process), aminoacyl-transfer RNA synthetase Class II (InterPro), and RSRFC4 (MEF2A) and NKX3A (both Transfac). We were intrigued by those concepts that referred to aminoacyl tRNA synthetases (KEGG pathway: http://www.genome.jp/kegg/ [24], [23]), as they potentially reflect an elevation in amino acid utilization and thus an increase in downstream protein biosynthesis upon androgen treatment (Figure 4, blue colored edges). This observation is in agreement with our earlier gene expression-based prediction of increased protein biosynthesis as one of the hallmarks during prostate cancer development [17]. Elevated aaRS activity was also among the significant concepts enriched by matched transcriptome data for androgen treated LNCaP cells (data not shown). Thus, such observations led us to select the aminoacyl tRNA synthetase concept for further analyses.

### Androgen regulation of aminoacyl tRNA synthetases

**Figure 5:**
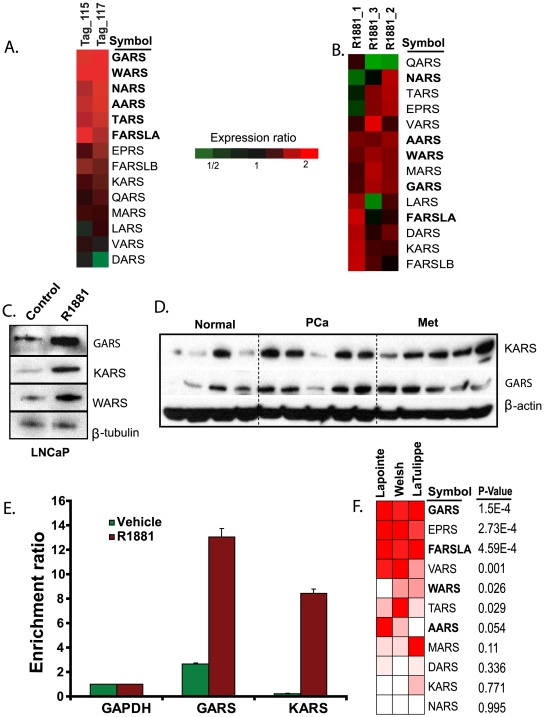
Androgen dependent up-regulation of aminoacyl tRNA synthetases in LNCaP, and their expression in prostate cancer. A) Heat map showing the elevated expression of aminoacyl-tRNA synthetases identified from iTRAQ experiment. Only proteins in bold face are designated as androgen up-regulated in the iTRAQ data based on the threshold cut-off used. B) Heat map showing the concordant over-expression of transcripts measured using oligonucleotide microarray (Affymetrix U133 Plus 2.0) for aminoacyl-tRNA synthetases shown in A. C) Immunoblot analysis of aminoacyl t-RNA synthetases GARS, KARS, and WARS in response to androgen treatment in LNCaP cells. Beta-tubulin is used as loading control. D) Immunoblot analysis of KARS and GARS in localized prostate cancer (PCa), and metastatic prostate cancer (Met) compared to normal (benign) prostate tissues. Beta-actin is used as loading control. E) Promoter-occupancy of AR on aminoacyl tRNA genes. Chromatin immunoprecipitation (ChIP) -PCR analysis shows the androgen-dependent enrichment of AR-binding to the target promoters of *GARS* and *KARS*. Enrichment ratio was calculated based on the amount of target amplification against the input DNA, with primers for *GAPDH* promoter used as control. The primer sequences spanning the gene promoters are given in Table S5. F) Meta-analysis of the aminoacyl t-RNA synthetase genes (of proteins shown in A) across three prostate cancer gene expression profiling studies. Oncomine heat map view showing the over-expression of a subset of aminoacyl-tRNA synthetase genes in localized prostate cancer compared to their benign controls. Gene symbols of proteins that pass through the cut-off value and were assigned as androgen up-regulated in iTRAQ data are indicated in bold face. P-value represents the significance of expression in two of the three studies. Red, white, and blue (not present in the figure) indicates relative over, unchanged, and under expression, respectively.

There were 7 proteins in the aaRS category that exceeded the set threshold in the combined list of proteins defined using both MS platforms: aaRS for alanine (AARS), phenylalanine (FARSLA), glycine (GARS), histidine (HARS), asparagine (NARS), threonine (TARS) and tryptophan (WARS). Of these only AARS and HARS were nominated by the MudPIT platform (Table S3) while AARS and the rest were nominated by the iTRAQ study (Table S1). Although, the total numbers of aaRS identified in iTRAQ and MudPIT experiments were 14 and 13, respectively, several of these proteins were identified with less than four peptides in MudPIT experiment making it less reliable for their spectral count based quantitation. This also indicates the low abundant nature of this critical class of proteins. Therefore, we focused to further analyze the iTRAQ identified aaRSs and examined if there is compression in expression ratio despite their elevated expression. Of the 14 aaRSs identified in the iTRAQ study, six had iTRAQ ratios 1.2 and above, and three of them had ratios 1.1 and above in both androgen-treated (115 and 117) replicate samples. Over-expression of majority these (nine of 14) aaRSs is depicted in a heat map (Figure 5A). A subset of these iTRAQ identified aaRSs (GARS, KARS, and WARS) were validated using androgen-treated LNCaP cells by immunoblot analysis (Figure 5C). Further, we examined their transcript levels using matched gene expression data for androgen-treated LNCaP cells (see Materials and Methods section). Nine of the fourteen proteins identified by our protein profiling were elevated at the transcript level across independent gene expression experiments (Figure 5B). This revealed potential regulation of these proteins by transcriptional activation by androgen. These data also support the observation of compression in fold change of protein expression in iTRAQ ratios.

Androgens mediate their positive effect on transcription by binding to androgen receptor, which in turn binds to androgen response elements on the promoters of target genes [25]. Thus, we proceeded to examine androgen binding to the promoters of aminoacyl tRNA synthetases using chromatin immunoprecipitation (ChIP, see Materials and Methods). Figure 5E shows representative example of increased occupancy of AR on the promoter of *GARS* and *KARS* confirming androgen regulation of its expression at the transcript level.

We next extended our cell line observation to prostate cancer tissue specimens. First, we used the ONCOMINE database (www.oncomine.org) to perform a meta-analysis for aminoacyl tRNA synthetase transcripts across multiple prostate cancer studies [26]. A subset of aaRSs was found to be up-regulated in localized prostate cancer samples in more than one study (Figure 5F). This observation was confirmed using immunoblot analysis of prostate-derived samples that included benign adjacent, localized prostate cancer, and metastatic disease. Importantly, four of the five localized PCa and four of the five metastatic PCa showed elevated expression of KARS and GARS when compared to three of the four benign samples tested (Figure 5D). These lend credence to a possible role of androgen-regulated aminoacyl tRNA synthetases action during prostate cancer progression.

## Discussion

To delineate the biological processes and pathways that are dysregulated by androgen in prostate cancer, we adopted a strategy of global MS-based proteomic profiling coupled to enrichment-based pathway mapping in the androgen-responsive prostate cancer cell line LNCaP. We profiled the proteome of LNCaP cells with and without androgen treatment using two proteomic platforms: quantitative iTRAQ MALDI-TOF MS and semi-quantitative MudPIT Ion Trap MS. Each of the platforms independently identified in excess of 850 high confidence proteins, yielding a total of 1306. The gain in cumulative protein counts is consistent with earlier reports describing the advantage of interrogating global proteomes using complementary mass spectrometry platforms [21], [27]. We were able to identify 126 proteins that were up-regulated upon androgen treatment. Included were known targets of androgen action: KLK3, FKBP5, FASN, AGR2 (anterior gradient homolog 2), SORD (sorbitol dehydrogenase), NDRG1, ACSL3 and FOLH1 (folate hydrolase 1). Additionally, nested in the compendia of differentially regulated proteins, were novel targets of androgen action that included PCID1 (PCI domain containing 1), RAB10 (ras-related GTP-binding protein RAB10), and PEA15 (phosphoprotein enriched in astrocytes 15).

An additional advantage of using the ion trap- based profiling strategy in tandem with TOF-TOF based quantitative profiling was that data from the former helped offset the loss of resolution resulting from iTRAQ ratio compression seen in the latter. This permitted the use of lower threshold level from iTRAQ-based quantitation for perturbation analysis. The ratio compression has been widely reported by others, e.g. [28], and is well illustrated by the direct comparison of iTRAQ ratios for androgen-regulated proteins with the corresponding expression as assessed by immunoblot analysis (Figure 3). As an example, protein FASN was identified with 17 peptides; among them the most differentially expressed peptide had an iTRAQ ratio of 2.79. Notably, averaging all iTRAQ ratios for 17 peptides resulted in a further compressed protein iTRAQ ratio of 1.85, while densitometry-based analysis of its expression by immunoblot revealed fold change of eight and the immunofluorescence analysis also showed a highly elevated expression. This conclusion is supported by the spectral counting of the MudPIT data, wherein FASN was represented by 417 spectra in the androgen-treated sample compared to only 45 spectra in the control sample. Such ratio compression, although seen in other studies, was more prominent in our iTRAQ MALDI- TOF/TOF data compared to other quantitative MS platforms such as ICAT [9]. This difference may be explained by a wider mass tolerance window for selection of peptide ions for MS/MS fragmentation in the ABI 4800 TOF/TOF mass analyzer used here [29]. We believe that this compression in ratio could partly explain the poor overlap between the sets of androgen-regulated proteins nominated from each of the two MS platforms. Additionally, factors such as differences in peptide processing (labeled versus unlabeled), peptide fractionation (on-line and offline), MS data acquisition (ion trap versus TOF/TOF mass analyzer), data normalization (ratios of spectral counts versus ratios of iTRAQ label peak intensities), and the properties of peptides selected for MS/MS sequencing could play pivotal roles in the lack of global concordance. These factors, in addition to the biological variations such as the multiple splice forms for a single accession could also have contributed to those three proteins that showed discordant trends between the two platforms observed.

Indeed, it has been established that different proteomic platforms show substantial differences in the physico-chemical properties of the identified peptides [30]. Thus, the strategy of using two different mass spectrometers, which differ both in their ionization source and principle of peptide detection, allowed us to build an additive list of proteins and gain deeper insight into the androgen-regulated proteome. With higher level enrichment analyses, our study showed that subsets of proteins, nominated by each of the two platforms, map to similar concepts including those that are androgen-derived and prostate cancer-derived. With a stringent threshold setting for the spectral count based semi-quantitation, the MudPIT experiment had nominated only two aaRSs candidates as androgen regulated. However, due to the nomination of higher number of aaRSs from iTRAQ experiment, molecular concept network with combined data set showed a set of interconnected aaRS concepts with significant scores (Figure 4). Even more intriguing, however, was that each of the two independent lists of proteins, when analyzed separately using MCM analysis, enriched for concepts that were known to be associated with prostate cancer progression but were not identified in the analysis of the combined dataset (Figure 4, Supplementary Figure S2). An example of this was the enrichment of ETS (ETV1 and ERG) over-expression concepts by the androgen-regulated proteins nominated by MudPIT analysis. This was interesting in the context of earlier reports that describe the existence of recurrent gene fusions involving ETS transcription factors to be pathonomonic to prostate cancer development [31], [32]. These gene fusions have been shown to bring ETS transcription factors under androgen control, and ETV1 (chromosome 7p21) is known to be over-expressed by androgen through its rearrangement to 14q13.3–14q21.1 region of chromosome 14 in LNCaP cell line studied here [31]. As the number of ETS regulated proteins identified in MudPIT was sufficient to map the ETS over-expression concept, lack of similar proportion of this class of proteins in iTRAQ platform possibly resulted in the disappearance of the same concept in the combined analysis. Thus, use of multiple mass spectrometric platforms helps to elucidate multiple roles of androgen action.

In addition to previously known androgen-regulated biological processes associated with prostate cancer progression, such as NDRG1 [33] that helped to validate our proteomic study, our list of androgen-regulated proteins was enriched for aminoacyl tRNA synthetases. Several aaRSs that are known to play a pivotal role in protein biosynthesis were included in this concept. This finding was interesting in the context of earlier gene expression-based predictions from our laboratory: an increase in protein synthesis during prostate cancer progression driven by actions of ETS transcription factors [17]. These observations lead us to the validation of this finding at multiple levels; starting at the level of regulatory controls with a combination of matched gene expression and chromatin immunoprecipitation assays. Finally, we used Western for orthogonal verification of the MS findings on the LNCaP cells, and showed that indeed the class of aaRSs are altered both at the transcript and protein level in prostate cancer.

## Materials and Methods

### Materials

Synthetic androgen R1881 (methyltrienolone) was purchased from Perkin Elmer (Boston, MA). A 10 mM R1881 stock was prepared in 100% ethanol and stored in −20°C. RPMI 1640 medium and fetal bovine serum was purchased from Invitrogen (Carlsbad, CA). Sequencing grade modified trypsin was from Promega (Madison, WI). Sample preparation reagents formic acid, trifluoroacetic acid, and HPLC solvents were from Fisher Scientific (Waltham, MA). Mass spectrometriy grade water was from Burdick and Jackson (Muskegon, MI, USA). Antibodies used were: KLK3 (kallikrein 3, rabbit polyclonal, DakoCytomation Carpinteria, CA ), NDRG1 (N-myc downstream regulated 1, goat polyclonal, Santa Cruz Biotechnology Inc., Santa Cruz, CA.), FASN (fatty acid synthase, mouse monoclonal, BD Transduction Labs, Franklin Lakes, NJ), FKBP5 (FK506 binding protein 5, mouse monoclonal, BD Transduction Labs), AR (mouse monoclonal-AR441, Lab Vision, Fremont, CA), GARS (glycyl-tRNA synthetase, rabbit polyclonal, Abcam, Cambridge, MA), WARS (tryptophanyl-tRNA synthetase, rabbit polyclonal, Abcam), KARS (lysyl-tRNA synthetase, rabbit polyclonal, Bethyl Laboratories, Montgomery, TX), β-tubulin (rabbit polyclonal, Santa Cruz), β-actin (rabbit polyclonal, Cell Signaling Technology, Inc., Danvers, MA). LNCaP cells were purchased from American Type Culture Collection (University Boulevard, Manassas, VA). Prostate specimens comprising adjacent normal, localized prostate cancer, and metastasis tissue utilized for immunoblot experiments were from the University of Michigan Rapid Autopsy Program which is part of University of Michigan Prostate Cancer Specialized Program of Research Excellence (SPORE) tissue core.

### Cell culture and sample preparation

LNCaP (ATCC number: CRL-1740™) cells were grown to 70% confluence in RPMI 1640 medium containing 10% FBS under 5% CO2 and 90% humidity. Phenol-red free RPMI 1640 supplemented with charcoal-stripped FBS was used to deplete endogenous androgen for two days. R1881, solubilized in ethanol, was added at a final concentration of 1 nM for 48 h, and ethanol was used as vehicle control in parallel experiments. Cells were washed with tris-buffered saline (TBS) and harvested using a cell scraper. **MudPIT:** cells were lysed in RapiGest™ SF cell lysis buffer as per the manufacturer's procedure (Waters Corporation, Milford, MA). Protein concentrations in the supernatant were estimated using Bradford assay (BioRad Laboratories, Hercules, CA). Proteins were reduced using 2 mM tris(2-carboxyethyl) phosphine hydrochloride at 60°C and alkylated by 5 mM Iodoacetamide at 37°C. Sequencing grade trypsin (Promega, Madison, WI), at the ratio of 1∶50, was added for digestion over-night at 37°C. The **iTRAQ protocol was as follows:** cells were lysed in a lysis solution containing 7 M urea, 2 M thiourea and 100 mM n- octyl-β-D-glucopyranoside, then mixed by intermittent vortexing over 20 min at room temperature, followed by centrifugation at 12000 RPM for 15 min. Protein concentrations in the supernatant were estimated using Bradford assay (BioRad Laboratories) and subjected to acetone precipitation before digestion. A 100 µg aliquot from each sample was used for the precipitation. Digestion and labeling using isobaric reagents (iTRAQ® Reagents Multiplex Kit, Applied Biosystems, Foster City, CA) were performed following the protocol described previously [28]. Eighty micrograms each of these biological, duplicate control samples and R1881-treated samples were labeled with different isobaric tags (114: control_1, 116: control_2, 115: R1881_1, 117: R1881_2). All four samples were combined and subjected to fractionation by SCX and RP-LC MALDI plating.

### Mass-spectrometry

#### Online 2D LC-MS/MS (MudPIT) of non-labeled tryptic peptides using LTQ

Paradigm MG4 HPLC system (Michrom Bioresources, Inc., Auburn, CA) was used for the separation of the peptides through a strong cation exchange trap and a reverse phase C18 microtrap (Michrom Bioresources, Inc.). Progressively increasing concentrations of ammonium formate (1.75 mM to 500 mM) were used for stepwise elution, and the trapped peptide salts at the microtrap were washed with buffer A (5% ACN, 0.0025% HFBA, 95% H20). The peptides were eluted using a 60 minute gradient starting with 5% ACN to 70% ACN with a two buffer system (Buffer A: as above; Buffer B: 95% ACN, 0.0025% HFBA, 5% H2O). The C-18 microtrap was coupled to an analytical C-18 column with a pulled tip (75 µm i.d., 10 cm length; New Objective Inc, Woburn, MA). Peptides were sprayed directly into the LTQ Mass Spectrometer (Thermo Fisher Corporation, Waltham, MA).

Equal amounts of proteins (7.5, 15, and 50 µg each) from either control or R1881-treated LNCaP cells were trypsinized as described above and purified using a desalting peptide macrotrap (Michrom Bioresources, Inc.) prior to online SCX and C18 separation. LC-MS/MS was performed for each sample in data dependent mode using Xcalibur software (Thermo Fisher Corporation). Each MS full scan (m/z = 400–2000) was collected with an average of 10 micro scans, and the three most abundant ions were subjected to fragmentation (MS/MS). Collision energy was set to 30% and with dynamic exclusion duration of 3 minutes.

#### Offline 2D LC-MS/MS of iTRAQ labeled peptides. SCX fractionation

Four iTRAQ-labeled samples were combined and fractionated on 2 sulfoethyl aspartamide SCX spin columns (SEM HIL-SCX, PolyLC, The Nest Group, Inc. Southboro, MA) equilibrated with 10 mM KH2PO4, (pH 4.5), 20 % ACN and run in parallel. Peptides were eluted with 50 µl each of 14 salt steps (35 to 500 mM KCl) and the paired eluates were combined and dried. **RP LC MALDI:** **A** Zorbax 300SB-C18 (3.5 um, 150×0.1 mm) column was used to fractionate each of the above listed fractions. A 90 min gradient method with a flow rate of 0.4 µl/min was employed (1100 series nanoflow LC system, Agilent Technologies, Santa Clara, CA). Column effluent was mixed (micro Tee, Agilent) with matrix (2.5 mg/ml α-CHCA and 10 mM NH_4_H_2_PO_4_ in methanol∶ isopropanol∶ ACN∶ H_2_O∶ acetic acid (14∶30∶22∶33∶0.6) delivered with an infusion pump (PHD200, Harvard Apparatus, Holliston, MA) at 0.9 µl/min and directly spotted (microFC, Agilent) at 0.42 min intervals onto a stainless steel MALDI target plate from 12 to 92 minutes (192 wells/plate, Applied Biosystems, Foster City, CA). **MALDI- TOF MS/MS analysis:** Data were acquired using 4800 Proteomics Analyzer -TOF/TOF™ (Applied Biosystems) linked to 4000 Series Explorer software (v. 3.0). Spectra from m/z 800–3500 were acquired using 750 laser shots. Spectra with signal to noise (S/N) of 120 were selected for MS/MS by applying 750–6000 laser shots. Atmospheric gas was used as the collision gas with a pressure of ∼6×10^-7^ Torr and collision energy of 1 kV. A seven point Gaussian smoothing and S/N of 15 were applied using cluster area S/N optimization for peak detection. Instrument default calibration was updated using monoisotopic masses for angiotensin I (m/z 1296.685), Glu1-fibrinopeptide B (m/z 1570.677), ACTH (18–39) (m/z 2465.199), des-Arg1-bradykinin (m/z 904.468), ACTH (1–17) (m/z 2093.087) and ACTH (7–38) (m/z 3657.923) (ABI).

### RNA Isolation and Microarray Analysis

Total RNA isolation was performed using TRIZOL reagent per the manufacturer's instructions (Invitrogen). Briefly, cells were washed with PBS (Phosphate buffer saline) and scraped in TRIZOL reagent. Approximately 250 µl of chloroform was added to 1 ml of sample and mixed by inversion. The sample was centrifuged at 13,000 rpm for 15 min at 4°C. Approximately 500 µl of isopropyl alcohol was added to supernatant and centrifuged at 13,000 rpm for 15 min at 4°C. The pellet was washed with 70% ethanol, and centrifuged at 13,000 for 10 minutes at 4°C. After ethanol removal, RNA was dried and dissolved in RNAse- and DNAse-free water. The RNA was subsequently purified using RNeasy mini kit (QIAGEN Inc., Valencia, CA). The amount and integrity of purified RNA was checked by NanoDrop ND-1000 Spectrophotometer (NanoDrop Technologies, Wilmington, DE) and Agilent Bioanalyzer (Agilent Technologies). Total RNA from the individual samples was analyzed on Affymetrix U133 Plus 2.0 arrays (Affymetrix, Inc., Santa Clara, CA). Complementary DNA synthesis, cRNA synthesis, hybridization, washing, and scanning were done following the manufacturer's protocols (Affymetrix, Inc.). The experimental details and raw data have been deposited in the NCBI Gene Expression Omnibus (GEO, http://www.ncbi.nlm.nih.gov/geo) and are accessible through GEO Series accession number GSE17044.

### Mass-spectrometry data analysis

#### MudPIT ESI- Ion Trap MS/MS

Raw files were converted into mzXML files and peptides were assigned to MS/MS spectra using SEQUEST [18] search against the Human IPI database version 3.24. The following search parameters were selected: 3 da precursor mass tolerance, average mass, semi-tryptic search with two or fewer missed cleavages, and oxidized methionine as a variable modification. In total, 120290 search results were obtained in the R1881 treated data set (32178, 47810, 39793, and 509 assignments to spectra of 1+, 2+, 3+, and 4+ charged peptide ions, respectively) and 113925 in the control data set (31643, 44,772, 36699, and 811 assignments to spectra of 1+, 2+, 3+, and 4+ charged peptide ions, respectively). Peptide assignments were validated using PeptideProphet [19], and the protein inference performed using ProteinProphet [20], as these tools are freely available as a part of the Trans-Proteomic Pipeline (www.proteomecenter.org). The list of protein identifications was filtered using a 0.9 probability threshold, which corresponds to less than 1% estimated false discovery rate (FDR). MS/MS data sets acquired on control and R1881 treated LNCaP cells were analyzed separately, resulting in 670 and 660 protein groups in each set (with indistinguishable protein accession numbers collapsed into a single group), respectively.

#### iTRAQ MALDI- TOF MS/MS analysis

MS/MS spectra were extracted from the raw data in Mascot Generic File format and converted to mzXML using IP Framework (www.proteomecommons.org). The mzXML files were searched using SEQUEST against the Human IPI database version 3.24 appended with an equal number of decoy sequences (reversed sequences from the original database). The following search parameters were selected: 0.5 da precursor mass tolerance, monoisotopic mass, semi-tryptic search with two or fewer missed cleavages, oxidized methionine, deamidation (Gln, Asn), iTRAQ label on Tyr, iTRAQ label on Lys and at the peptide N-terminus, and thiomethyl cysteine were specified as variable modifications. In total, 8580 SEQUEST search results (all from singly-charged spectra) were obtained and further processed using PeptideProphet and ProteinProphet, leading to the identification of 3686 peptides mapping to 904 proteins. Of these, 3550 peptides were quantified with post-data normalization, which mapped to 879 proteins. The estimated FDR using target-decoy strategy was below 0.5% [13].

### Statistical analysis of differential protein expression

#### iTRAQ analysis

Double-duplex iTRAQ experiment consisting of duplicates of vehicle- and R1881-treated samples resulted in the identification of 3686 peptides as shown above. Quantitative information for each peptide identified was extracted from the MS/MS spectrum using Libra, available as a part of the Trans-Proteomic Pipeline. Based on the distribution pattern of peak areas of tags 114, 115, 116 and 117 for all the peptides, a median centered normalization was applied at the peptide level to achieve a mean close to unity. Peptides with no value for peak area (peak intensity below a default threshold in Libra) in any of the tagged channels were removed. Outlier peptides that had iTRAQ values three standard deviations (SD) away from the population median were considered abnormal and were removed from controls (114 vs. 116) or treatments (115 vs. 117). This procedure resulted in a final set of 3550 peptides mapping to 879 proteins. The relative protein expression ratios were then determined with respect to the first control sample (tag 114). Intensities of peaks at 115, 116 and 117 were divided by the 114 peak intensity, and the ratios of all peptides corresponding to the same proteins were averaged. The resulting protein ratios were again normalized by their population median. The standard deviation of the control replicate (tag 116, SD = 0.16) was used as a scale to set the threshold ratio for differentially expressed proteins. A minimum threshold of 1.25 SD (ratio of 1.2 for up-regulated and 0.83 for down-regulated) from the global mean in both androgen-treated samples and not less than 1.5 SD in one of the androgen treated samples (1.25 and 0.8 for up- and down-regulated, respectively) were considered as androgen regulated.

#### MudPIT analysis

Proteins identified in the two separate mass spectrometry runs (control vs. androgen treated) were combined into a single list, and ambiguities resulting from protein isoforms and multiple accession numbers [34] were resolved using in-house software. For each protein in the combined list (or protein group with multiple accession numbers), the total number of MS/MS spectra, which were assigned to peptides was calculated. The spectrum count measures for each protein were normalized. The normalization was done to account for small differences in the number of identified peptides in each analysis. Normalization factor was taken as the total number of spectra observed in each experiment that were assigned a peptide, from a protein identified in both experiments with high probability. For proteins that were identified in both experiments, the ratio of normalized spectrum counts was calculated and then log-transformed. The resulting distribution was fitted using a robust Gaussian distribution fitting procedure with outlier removal (10% of the data), and the mean and standard deviation (SD) were determined. Two-SD threshold was applied to derive the list of differentially expressed proteins. Consequently, the final established requirement was that, if the protein were identified by a single MS/MS spectrum in one of the experiments, it should be identified by four or more spectra in the other experiment. Similarly, a threshold of four or more spectra was applied to proteins identified in one of the runs only.

#### Western

Proteins for immunoblotting were resolved by 4–12% NUPAGE gels (Invitrogen) and transferred to PVDF membranes (GE Healthcare Bio-Sciences Corp., Piscataway, NJ). Membranes were then blocked with 5% skimmed milk in TBS-T (20 mM Tris.Cl, pH 7.4, 150 mM NaCl, 0.1 % Tween 20) over-night. Antibodies (indicated in ‘Materials’) were added in TBS-T containing 2.5% skimmed milk and the blots were washed with TBST (TBS +0.1% Tween 20). Immunoblot signals were developed using enhanced chemiluminescence reagent (ECL plus, GE Healthcare Bio-Sciences Corp., Piscataway, NJ). Western band intensity ratios were calculated using the ‘ImageJ’ software version 1.38 (rsb.info.nih.gov/ij/)

#### Immunofluorescence and confocal microscopy

Control and R1881 treated cells were fixed on glass slides. Slides were heated in citrate buffer (pH 6.0) for 15 minutes in a pressure cooker. The slides were then blocked in PBS-T with 5% normal donkey serum for 1 hour. A mixture of rabbit anti-AR antibody (Labvision, Fremont, CA) and mouse anti-FASN antibody (BD Transduction Labs, Franklin Lakes, NJ) was added to the slides and incubated overnight at 4°C. Slides were then incubated with secondary antibodies (anti-rabbit Alexa 555 and anti-mouse Alexa-486 (Invitrogen) at 1∶1000 dilution for 1 hour. They were washed and then mounted using Vectashield mounting medium. Confocal images were taken with a Ziess LSM510 META imaging system using Argon and Helium Neon 1 and Helium Neon 2 light sources (Carl Zeiss, Thornwood, NY). The wavelengths used were 486 nm and 555 nm for Alexa-486 and Alexa-555, respectively. Color images were exported as TIFF images.

#### Oncomine and Molecular Concepts Mapping analyses

Methods used to identify gene signatures in the Molecular Concepts Mapping (MCM) tool are described elsewhere [23]. A“molecular concept” is defined as a set of genes or proteins specific to a biological process or a regulatory mechanism or a particular disease represented by previously well characterized data sets. Currently, MCM has a total of 13364 such concepts consisting of 17 types that are derived from 13 sources including the prostate cancer gene expression signatures from Oncomine database [26]. For our analysis we used all concept types except the ‘Connectivity map’, and ‘Oncomine clusters’ [26]. All of our protein data sets were loaded into Oncomine, and analyzed through the Oncomine linked MCM [23], [26]. MCM computes Fisher's exact test for each pair of molecular concepts for association. All results that had an odds ratio greater than 1.25 and p-value of less than 0.01 were stored. Network maps were obtained between the selected enriched concepts in our protein sets and visualized using the MCM. From Oncomine ‘Cancer Signature’ type concepts, only the top 1%, 5% or 10% gene set concepts were included in our networks. For network analysis, we selected the most significant concept, at least one most significant concept from each concept type that was associated, and any concept that showed relevance to prostate cancer or androgen treatment.

Meta-analysis of our proteomics data and the prostate cancer tissue microarray data was performed using MCM [26], and the heat map was visualized with the genes ranked based on their P- values. Three data sets used for the heat map were: 1) Lappointe (normal prostate, 41 samples vs. prostate cancer, 62 samples); 2) Welsh (normal prostate, 9 samples vs. prostate cancer, 25 samples); 3) LaTulippe (non-neoplastic prostate, 3 samples vs. prostate carconima, 23 samples).

#### Chromatin immunoprecipitation and PCR

Chromatin immunoprecipitation was performed according to the published protocol [35]. Polyclonal antibody to AR (PG-21, Upstate, NY, USA) was used for immunoprecipitation on the control (vehicle treated) and androgen treated LNCaP cell nuclear lysates. For PCR analysis of target gene enrichment analysis, 2 µl of input DNA, and target-enriched DNA were used. PCR primer sequences designed to flank the target gene promoters and used for ChIP-PCR are given in Supplementary Table S5.

#### Supporting Information

Two supplementary figures, and five supplementary tables. This material is available free at http://pubs.plosone.org.

## Supporting Information

Figure S1 (0.27 MB PNG)

Figure S2 (0.76 MB PNG)

Table S1 (0.01 MB PDF)

Table S2 (0.01 MB PDF)

Table S3 (0.01 MB PDF)

Table S4 (0.01 MB PDF)

Table S5 (0.01 MB PDF)
