# Supplementary figures and images for: Proteomic Interrogation of Androgen Action in Prostate Cancer Cells Reveals Roles of Aminoacyl tRNA Synthetases
Source: PLoS One. 2009 Sep 18;4(9):e7075. doi: 10.1371/journal.pone.0007075 (PMC2740864; doi:10.1371/journal.pone.0007075)

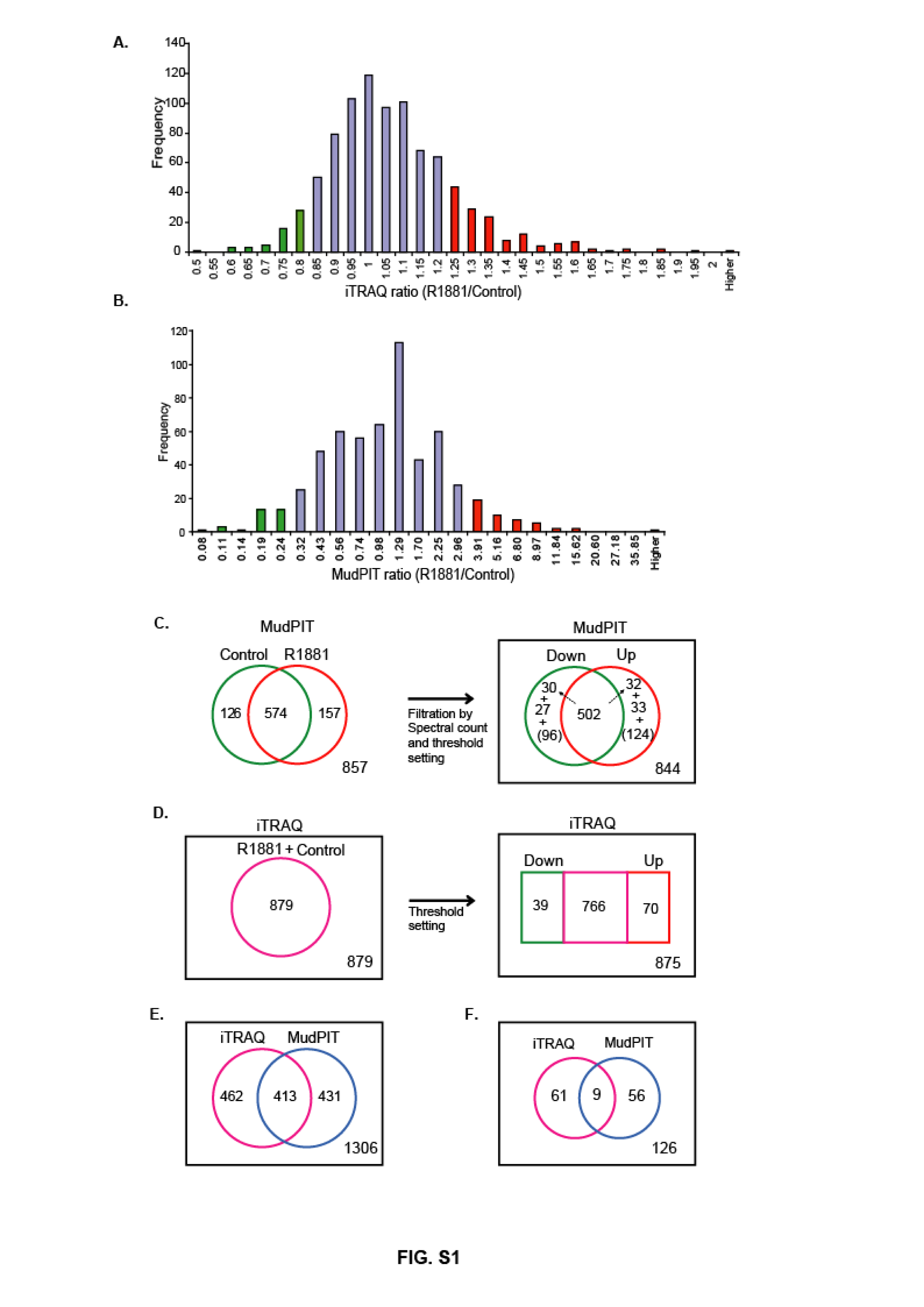

Supplement: Figure S1 — (0.27 MB PNG) [file pone.0007075.s001.png]

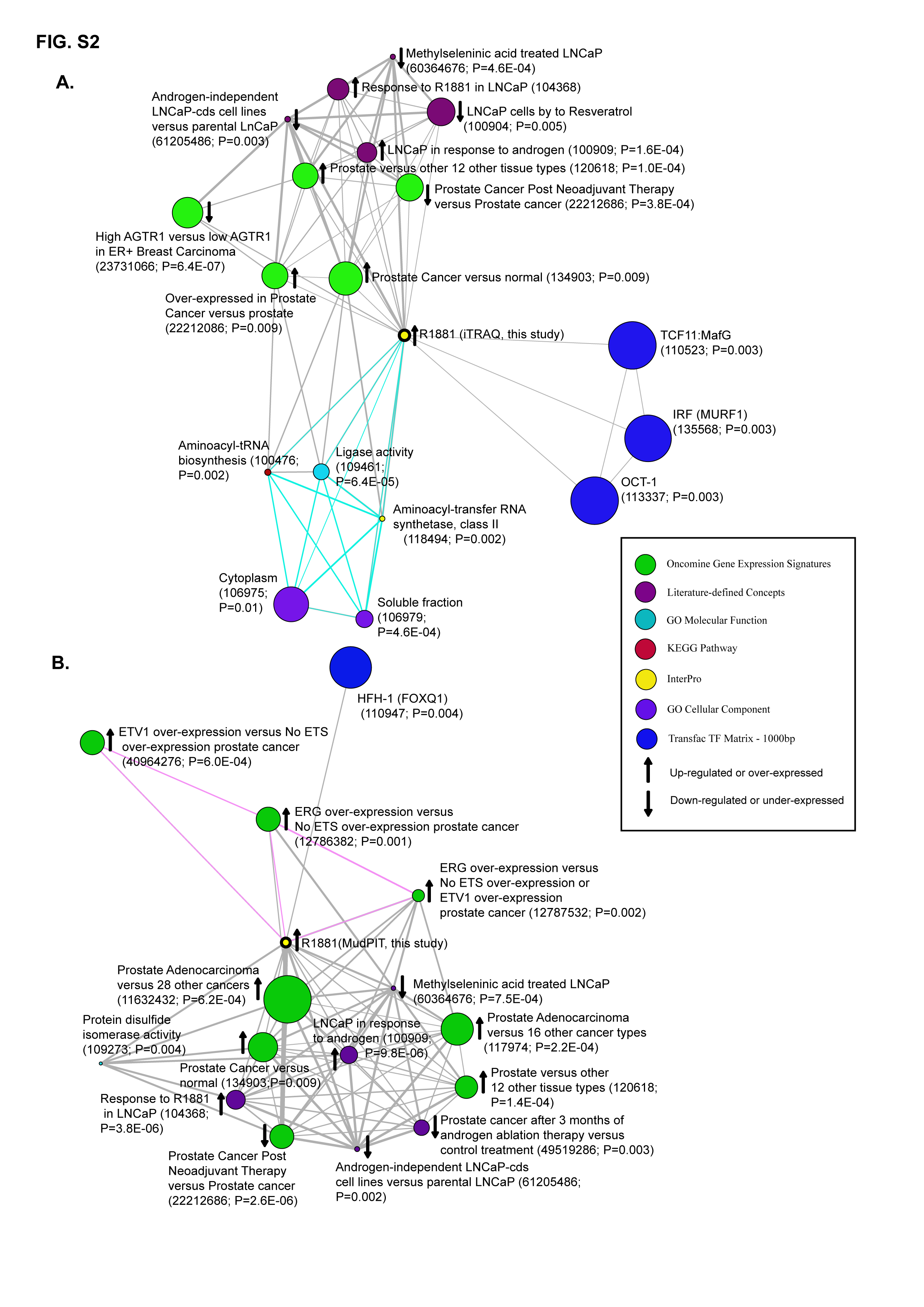

Supplement: Figure S2 — (0.76 MB PNG) [file pone.0007075.s002.png]
