# Supplementary material for: Proteomic Interrogation of Androgen Action in Prostate Cancer Cells Reveals Roles of Aminoacyl tRNA Synthetases
Source: PLoS One. 2009 Sep 18;4(9):e7075. doi: 10.1371/journal.pone.0007075 (PMC2740864; doi:10.1371/journal.pone.0007075)
Supplement: Table S1 — (0.01 MB PDF) [file pone.0007075.s003.pdf]

Table S1 Proteins identified as androgen up-regulated through iTRAQ LC-MS/MS

IPI_ID	Entrez_ ID	Symbol	Description	MS	P	Ratio _114	Ratio_ 116	Ratio_ 115	Ratio _117
IPI00000001	6780	STAU1	Isoform Long of Double-stranded RNA-binding protein Staufen homolog 1	1	0.99	1	1.24	1.25	1.27
IPI00000495	10480	EIF3M	Eukaryotic translation initiation factor 3, subunit M	1	0.55	1	0.98	1.55	1.64
IPI00000684	6675	UAP1	Isoform AGX2 of UDP-N-acetylhexosamine pyrophosphorylase	1	0.98	1	0.87	1.74	1.66
IPI00001952	23052	ENDOD1	Endonuclease domain-containing 1 protein precursor	1	0.99	1	1.16	1.51	1.64
IPI00002519	6470	SHMT1	Serine hydroxymethyltransferase, cytosolic	1	0.97	1	1.17	1.47	1.28
IPI00003783	5605	MAP2K2	Dual specificity mitogen-activated protein kinase kinase 2	1	0.98	1	1.27	1.50	1.79
IPI00003799	23593	HEBP2	Isoform 2 of Heme-binding protein 2	1	0.97	1	0.76	1.26	1.24
IPI00004358	5834	PYGB	Glycogen phosphorylase, brain form	6	1.00	1	0.88	1.36	1.30
IPI00005154	6749	SSRP1	FACT complex subunit SSRP1	2	0.97	1	1.26	1.31	1.34
IPI00008438	6204	RPS10	Ribosomal protein S10	15	1.00	1	1.13	1.36	1.31
IPI00008524	26986	PABPC1	Isoform 1 of Polyadenylate-binding protein 1	4	1.00	1	0.97	1.31	1.21
IPI00009315	64746	ACBD3	Golgi resident protein GCP60	1	1.00	1	1.00	1.28	1.24
IPI00010182	1622	DBI	Diazepam binding inhibitor	8	1.00	1	0.97	1.83	1.76
IPI00010471	3936	LCP1	Plastin-2	1	0.99	1	0.84	1.67	1.94
IPI00010858	354	KLK3	Prostate-specific antigen precursor	3	1.00	1	1.15	1.44	1.44
IPI00011253	6188	RPS3	40S ribosomal protein S3	1	0.99	1	0.78	1.27	1.46
IPI00011603	5709	PSMD3	26S proteasome non-ATPase regulatory subunit 3	2	1.00	1	0.92	1.24	1.39
IPI00012007	191	AHCY	Adenosylhomocysteinase	2	1.00	1	1.09	1.60	1.52
IPI00014850	8682	PEA15	Astrocytic phosphoprotein PEA-15	2	0.83	1	0.93	1.41	1.46
IPI00015029	10728	PTGES3	Prostaglandin E synthase 3	2	1.00	1	0.83	1.25	1.31
IPI00017376	10483	SEC23B	Protein transport protein Sec23B	1	0.99	1	0.79	1.57	1.30
IPI00017763	4676	NAP1L4	Nucleosome assembly protein 1-like 4	2	0.99	1	0.94	1.34	1.42
IPI00020944	2222	FDFT1	Squalene synthetase	1	0.99	1	1.28	1.74	2.01
IPI00021290	47	ACLY	ATP-citrate synthase	3	1.00	1	0.83	1.29	1.32
IPI00021347	7332	UBE2L3	Ubiquitin-conjugating enzyme E2 L3	2	0.99	1	0.80	1.29	1.47

IPI00022078	10397	NDRG1	N-myc downstream regulated	8	1.00	1	1.06	1.61	1.76
IPI00025084	826	CAPNS1	Calpain small subunit 1	2	0.98	1	1.05	1.60	1.48
IPI00026689	983	CDC2	Cell division cycle 2	1	0.98	1	1.16	1.40	1.55
IPI00026781	2194	FASN	Fatty acid synthase	59	1.00	1	0.99	1.85	1.78
IPI00027442	16	**AARS**	Alanyl-tRNA synthetase, cytoplasmic	6	1.00	1	1.00	1.29	1.23
IPI00030179	6129	RPL7	60S ribosomal protein L7	2	0.86	1	1.03	1.25	1.27
IPI00030706	10598	AHSA1	Activator of 90 kDa heat shock protein ATPase homolog 1	1	0.99	1	1.09	1.47	1.49
IPI00030876	1729	DIAPH1	Protein diaphanous homolog 1	1	0.97	1	0.99	1.32	1.28
IPI00031397	2181	ACSL3	Long-chain-fatty-acid--CoA ligase 3	5	1.00	1	1.06	2.45	2.76
IPI00031801	8531	CSDA	Cold shock domain protein A	1	0.82	1	0.87	1.30	1.31
IPI00031820	2193	**FARSLA**	Phenylalanyl-tRNA synthetase alpha chain	3	0.98	1	1.15	1.20	1.41
IPI00064765	140801	RPL10L	60S ribosomal protein L10-like	6	1.00	1	1.05	1.32	1.35
IPI00140420	27044	SND1	Staphylococcal nuclease domain-containing protein 1	6	1.00	1	0.90	1.26	1.30
IPI00156282	2873	GPS1	G protein pathway suppressor 1	1	0.98	1	1.38	1.60	1.40
IPI00176854	6231	RPS26	Ribosomal protein S26	4	1.00	1	1.01	1.28	1.30
IPI00185374	5718	PSMD12	26S proteasome non-ATPase regulatory subunit 12	1	0.98	1	0.86	1.56	1.20
IPI00186290	1938	EEF2	Elongation factor 2	12	1.00	1	0.95	1.30	1.40
IPI00215790	6169	RPL38	60S ribosomal protein L38	3	1.00	1	1.10	1.28	1.30
IPI00216057	6652	SORD	Sorbitol dehydrogenase	10	1.00	1	1.00	1.59	1.64
IPI00216105	29789	GTPBP9	Isoform 2 of Putative GTP-binding protein 9	1	0.91	1	1.20	1.53	1.63
IPI00216298	7295	TXN	Thioredoxin	3	1.00	1	0.87	1.41	1.30
IPI00216587	6202	RPS8	40S ribosomal protein S8	4	1.00	1	1.16	1.57	1.42
IPI00218775	2289	FKBP5	FK506-binding protein 5	3	1.00	1	1.09	1.91	1.91
IPI00220301	9588	PRDX6	Peroxiredoxin-6	17	1.00	1	0.92	1.24	1.28
IPI00220642	7532	YWHAG	14-3-3 protein gamma	1	0.99	1	0.90	1.30	1.28
IPI00221092	6217	RPS16	40S ribosomal protein S16	1	0.80	1	1.08	1.32	1.47
IPI00291419	39	ACAT2	Acetyl-CoA acetyltransferase, cytosolic	2	0.99	1	1.01	1.26	1.50
IPI00292020	6723	SRM	Spermidine synthase	1	0.99	1	0.75	1.21	1.47
IPI00295400	7453	**WARS**	Tryptophanyl-tRNA synthetase, cytoplasmic	1	0.99	1	0.82	1.33	1.36
IPI00295857	1314	COPA	Coatomer subunit alpha	5	1.00	1	1.16	1.29	1.20
IPI00296197	64374	SIL1	Nucleotide exchange factor SIL1 precursor	1	0.98	1	1.18	1.20	1.51
IPI00300127	55226	NAT10	N-acetyltransferase 10	2	1.00	1	1.21	1.22	1.38
IPI00305152	22872	SEC31A	SEC31 homolog A isoform 4	3	1.00	1	1.20	1.21	1.28

IPI00306960	4677	**NARS**	Asparaginyl-tRNA synthetase, cytoplasmic	4	1.00	1	0.91	1.29	1.20
IPI00328082	6897	**TARS**	Threonyl-tRNA synthetase	1	0.99	1	1.10	1.25	1.22
IPI00394699	497661	C18orf32	Chromosome 18 open reading frame 32	2	0.98	1	0.96	1.31	1.57
IPI00399212	389842	LOC389842	Similar to Ran-specific GTPase-activating protein	1	0.98	1	0.86	1.55	1.56
IPI00410693	26135	SERBP1	Plasminogen activator inhibitor 1 RNA-binding protein	6	1.00	1	1.07	1.31	1.20
IPI00411937	10528	NOL5A	Nucleolar protein Nop56	3	1.00	1	1.27	1.31	1.29
IPI00413451	5269	SERPINB6	Serpin peptidase inhibitor, clade B, member 6	1	0.99	1	1.03	1.45	1.49
IPI00465260	2617	**GARS**	Glycyl-tRNA synthetase	3	1.00	1	0.84	1.41	1.30
IPI00550364	55276	PGM2	Phosphoglucomutase-2	1	0.99	1	1.06	1.42	1.20
IPI00554777	440	ASNS	Asparagine synthetase	1	0.56	1	0.89	1.26	1.46
IPI00646689	84817	TXNL5	Thioredoxin-like protein 5	1	0.98	1	1.02	1.35	1.28
IPI00741181	644816	LOC644816	Similar to Heat shock protein HSP 90-beta	4	1.00	1	0.86	1.21	1.26

MS = Mass spectra, P = Protein probability
