# Supplementary material for: Proteomic Interrogation of Androgen Action in Prostate Cancer Cells Reveals Roles of Aminoacyl tRNA Synthetases
Source: PLoS One. 2009 Sep 18;4(9):e7075. doi: 10.1371/journal.pone.0007075 (PMC2740864; doi:10.1371/journal.pone.0007075)
Supplement: Table S2 — (0.01 MB PDF) [file pone.0007075.s004.pdf]

Table S2 Proteins identified as androgen down-regulated through iTRAQ LC-MS/MS

IPI_ID	Entrez_ ID	Symbol	Description	MS	P	Ratio _114	Ratio_ 116	Ratio_ 115	Ratio _117
IPI00642409	3107	HLA-C	HLA class I histocompatibility antigen, Cw-7 alpha chain precursor	1	0.92	1	1.10	0.48	0.60
IPI00012382	6626	SNRPA	U1 small nuclear ribonucleoprotein A	1	0.99	1	0.71	0.56	0.71
IPI00246058	10015	PDCD6IP	PDCD6IP protein	1	0.99	1	0.68	0.57	0.69
IPI00028376	1678	TIMM8A	Mitochondrial import inner membrane translocase subunit Tim8 A	1	0.95	1	1.06	0.57	0.41
IPI00376427	4685	NCAM2	Neural cell adhesion molecule 2 precursor	2	1.00	1	0.96	0.60	0.57
IPI00296141	29952	DPP7	Dipeptidyl-peptidase 2 precursor	1	0.98	1	0.94	0.61	0.71
IPI00465256	50808	AK3	GTP:AMP phosphotransferase mitochondrial	2	0.98	1	0.81	0.64	0.65
IPI00011229	1509	CTSD	Cathepsin D precursor	8	1.00	1	0.99	0.67	0.70
IPI00028514	2346	FOLH1	Isoform PSMA-1 of Glutamate carboxypeptidase 2	9	1.00	1	1.00	0.68	0.63
IPI00027438	10211	FLOT1	Flotillin-1	3	1.00	1	0.97	0.69	0.63
IPI00293464	1642	DDB1	DNA damage-binding protein 1	1	0.98	1	0.73	0.70	0.71
IPI00247063	4311	MME	Neprilysin	12	1.00	1	0.99	0.71	0.72
IPI00219034	4702	NDUFA8	NADH dehydrogenase [ubiquinone] 1 alpha subcomplex subunit 8	1	0.98	1	1.12	0.71	0.77
IPI00006723	9410	WDR57	WD repeat protein 57	1	0.77	1	0.86	0.71	0.73
IPI00003419	10944	C11orf58	Small acidic protein	1	0.98	1	0.94	0.72	0.78
IPI00016250	9513	FXR2	Fragile X mental retardation syndrome-related protein 2	1	0.97	1	0.78	0.72	0.56
IPI00414896	8635	RNASET2	Isoform 1 of Ribonuclease T2	1	0.82	1	1.04	0.73	0.77
IPI00029764	10946	SF3A3	Splicing factor 3A subunit 3	1	1.00	1	0.95	0.73	0.78
IPI00374065	375056	MIA3	similar to melanoma inhibitory activity 3 isoform 1	4	1.00	1	0.89	0.73	0.76
IPI00008868	4131	MAP1B	Microtubule-associated protein 1B	3	1.00	1	0.86	0.73	0.75
IPI00013891	29896	TRA2A	Isoform Long of Transformer-2 protein homolog	1	0.98	1	1.00	0.74	0.79
IPI00179529	481	ATP1B1	Isoform 1 of Sodium\potassium-transporting ATPase subunit beta-1	3	1.00	1	0.86	0.74	0.71
IPI00550020	5763	PTMS	Parathymosin	4	1.00	1	0.91	0.75	0.71
IPI00006663	217	ALDH2	Aldehyde dehydrogenase, mitochondrial precursor	1	0.99	1	1.01	0.75	0.73
IPI00020956	3068	HDGF	Hepatoma-derived growth factor	3	1.00	1	0.83	0.76	0.68
IPI00027834	3191	HNRPL	heterogeneous nuclear ribonucleoprotein L isoform a	4	1.00	1	0.86	0.76	0.78

IPI00759493	8802	SUCLG1	succinate-CoA ligase, GDP-forming, alpha subunit	1	1.00	1	0.98	0.77	0.73
IPI00029266	6635	SNRPE	Small nuclear ribonucleoprotein E	2	0.98	1	0.79	0.77	0.76
IPI00019018	7108	TM7SF2	Isoform 1 of Delta(14)-sterol reductase	1	0.99	1	1.44	0.77	0.78
IPI00002521	522	ATP5J	ATP synthase coupling factor 6, mitochondrial precursor	1	1.00	1	0.93	0.77	0.76
IPI00215884	6426	SFRS1	splicing factor, arginine\serine-rich 1	4	1.00	1	0.97	0.78	0.71
IPI00010740	6421	SFPQ	Isoform Long of Splicing factor, proline- and glutamine-rich	17	1.00	1	0.92	0.78	0.80
IPI00293303	5641	LGMN	Legumain precursor	1	0.99	1	1.12	0.78	0.82
IPI00182469	1500	CTNND1	Isoform 1AB of Catenin delta-1	6	1.00	1	1.01	0.80	0.79
IPI00026546	5049	PAFAH1B2	Platelet-activating factor acetylhydrolase IB subunit beta	2	1.00	1	0.58	0.80	0.60
IPI00026167	4809	NHP2L1	NHP2-like protein 1	1	0.98	1	0.80	0.80	0.77
IPI00304596	4841	NONO	Non-POU domain-containing octamer-binding protein	16	1.00	1	0.92	0.81	0.79
IPI00221354	2521	FUS	Isoform Short of RNA-binding protein FUS	4	1.00	1	0.88	0.82	0.77
IPI00008454	51726	DNAJB11	DnaJ homolog subfamily B member 11 precursor	1	0.88	1	1.07	0.82	0.59

MS = Mass spectra, P = Protein probability
