# Supplementary material for: Proteomic Interrogation of Androgen Action in Prostate Cancer Cells Reveals Roles of Aminoacyl tRNA Synthetases
Source: PLoS One. 2009 Sep 18;4(9):e7075. doi: 10.1371/journal.pone.0007075 (PMC2740864; doi:10.1371/journal.pone.0007075)
Supplement: Table S3 — (0.01 MB PDF) [file pone.0007075.s005.pdf]

**Table S3 Proteins identified as androgen up-regulated through MudPIT LC-MS/MS**

| IPI_ID | Entrez_ID | Symbol | Protein Description | MS_ R1881 | P_ R1881 | MS_ Control | P_ Control | Ratio (R1881/ Control) |
|---|---|---|---|---|---|---|---|---|
| IPI00031397 | 2181 | ACSL3 | Long-chain-fatty-acid--CoA ligase 3 | 45 | 1.00 | 1 | 0.56 | 47.30 |
| IPI00791902 | 51372 | CCDC72 | Coiled-coil domain-containing protein 72 | 12 | 1.00 | 1 | 0.72 | 12.61 |
| IPI00016513 | 10890 | RAB10 | Ras-related protein Rab-10 | 12 | 1.00 | 1 | 1.00 | 12.61 |
| IPI00012828 | 30 | ACAA1 | 3-ketoacyl-CoA thiolase, peroxisomal precursor | 10 | 1.00 | 1 | 0.98 | 10.51 |
| IPI00795589 | 2194 | FASN | Fatty acid synthase | 417 | 1.00 | 45 | 1.00 | 9.74 |
| IPI00140827 | 6612 | SUMO3 | Small ubiquitin-related modifier 3 precursor | 8 | 1.00 | 1 | 0.98 | 8.41 |
| IPI00219078 | 488 | ATP2A2 | ATPase, Ca++ transporting, cardiac muscle, slow twitch 2 | 8 | 1.00 | 1 | 0.67 | 8.41 |
| IPI00336094 | 3028 | HSD17B10 | hydroxysteroid (17-beta) dehydrogenase 10 | 7 | 1.00 | 1 | 0.96 | 7.36 |
| IPI00215948 | 1495 | CTNNA1 | Isoform 1 of Catenin alpha-1 | 7 | 1.00 | 1 | 0.97 | 7.36 |
| IPI00744135 | 6238 | RRBP1 | Isoform 1 of Ribosome-binding protein 1 | 13 | 1.00 | 2 | 0.99 | 6.83 |
| IPI00031131 | 57136 | C20orf3 | Chromosome 20 open reading | 6 | 1.00 | 1 | 1.00 | 6.31 |
| IPI00782968 | 7163 | TPD52 | Tumor protein D52 isoform 1 | 6 | 1.00 | 1 | 0.56 | 6.31 |
| IPI00009904 | 9601 | PDIA4 | Protein disulfide-isomerase A4 precursor | 83 | 1.00 | 15 | 1.00 | 5.82 |
| IPI00328748 | 7873 | ARMET | ARMET protein precursor | 11 | 1.00 | 2 | 0.99 | 5.78 |
| IPI00555610 | 79026 | AHNAK | Neuroblast differentiation-associated protein AHNAK | 5 | 1.00 | 1 | 0.85 | 5.26 |
| IPI00448095 | 51181 | DCXR | Dicarbonyl/L-xylulose reductase | 5 | 1.00 | 1 | 0.84 | 5.26 |
| IPI00024911 | 10961 | ERP29 | Endoplasmic reticulum protein ERp29 precursor | 5 | 1.00 | 1 | 1.00 | 5.26 |
| IPI00556589 | 650788 | LOC650788 | Similar to 40S ribosomal protein S28 | 4 | 0.99 | 1 | 1.00 | 4.20 |
| IPI00101186 | 23223 | RRP12 | Ribosomal RNA processing 12 homolog | 4 | 0.94 | 1 | 0.91 | 4.20 |
| IPI00220271 | 10327 | AKR1A1 | Aldo-keto reductase family 1, member A1 | 4 | 1.00 | 1 | 0.84 | 4.20 |
| IPI00218200 | 10134 | BCAP31 | B-cell receptor-associated protein 31 | 8 | 1.00 | 2 | 0.96 | 4.20 |
| IPI00478302 | 9537 | TP53I11 | Tumor protein p53 inducible protein 11 | 4 | 0.78 | 1 | 0.72 | 4.20 |
| IPI00413686 | 8943 | AP3D1 | Isoform 3 of AP-3 complex subunit delta-1 | 4 | 1.00 | 1 | 1.00 | 4.20 |
| IPI00028006 | 5690 | PSMB2 | Proteasome subunit beta type 2 | 4 | 0.99 | 1 | 1.00 | 4.20 |
| IPI00010706 | 2937 | GSS | Glutathione synthetase | 4 | 1.00 | 1 | 0.61 | 4.20 |
| IPI00022744 | 1434 | CSE1L | CSE1 chromosome segregation 1-like | 4 | 1.00 | 1 | 0.67 | 4.20 |
| IPI00787737 | 378 | ARF4 | ADP-ribosylation factor 4 | 8 | 1.00 | 2 | 1.00 | 4.20 |

| | | | | | | | | |
|---|---|---|---|---|---|---|---|---|
| IPI00162207 | 84626 | KRBA1 | KRAB-A domain containing 1 | 7 | 1.00 | 2 | 0.77 | 3.68 |
| IPI00219365 | 4478 | MSN | Moesin | 7 | 1.00 | 2 | 1.00 | 3.68 |
| IPI00215919 | 381 | ARF5 | ADP-ribosylation factor 5 | 7 | 1.00 | 2 | 1.00 | 3.68 |
| IPI00790740 | 2923 | PDIA3 | Protein disulfide-isomerase A3 precursor | 154 | 1.00 | 45 | 1.00 | 3.60 |
| IPI00784131 | 16 | AARS | Alanyl-tRNA synthetase, cytoplasmic | 13 | 1.00 | 4 | 1.00 | 3.42 |
| IPI00743671 | 2289 | FKBP5 | FK506-binding protein 5 | 17 | 1.00 | | | |
| IPI00025242 | 10720 | UGT2B11 | UDP-glucuronosyltransferase 2B11 precursor | 15 | 1.00 | | | |
| IPI00007427 | 10551 | AGR2 | Anterior gradient homolog 2 | 14 | 1.00 | | | |
| IPI00040297 | 354 | KLK3 | Prostate specific antigen isoform 3 preproprotein | 11 | 0.99 | | | |
| IPI00792844 | 27347 | STK39 | STE20\SPS1-related proline-alanine-rich protein kinase | 10 | 1.00 | | | |
| IPI00220663 | 3098 | HK1 | Isoform 2 of Hexokinase-1 | 9 | 1.00 | | | |
| IPI00001952 | 23052 | ENDOD1 | Endonuclease domain-containing 1 protein precursor | 8 | 1.00 | | | |
| IPI00397466 | 8720 | MBTPS1 | Membrane-bound transcription factor peptidase, site 1 | 7 | 1.00 | | | |
| IPI00798025 | 8209 | C21ORF33 | Chromosome 21 open reading frame 33 | 7 | 1.00 | | | |
| IPI00029744 | 6742 | SSBP1 | Single-stranded DNA-binding protein | 6 | 1.00 | | | |
| IPI00297037 | 8611 | PPAP2A | Phosphatidic acid phosphatase type 2A | 6 | 1.00 | | | |
| IPI00299084 | 55161 | TMEM33 | Transmembrane protein 33 | 6 | 1.00 | | | |
| IPI00607787 | 6675 | UAP1 | UDP-N-acetylhexosamine pyrophosphorylase | 6 | 1.00 | | | |
| IPI00007764 | 51155 | HN1 | Hematological and neurological expressed 1 isoform 1 | 5 | 0.94 | | | |
| IPI00016339 | 5878 | RAB5C | Ras-related protein Rab-5C | 5 | 1.00 | | | |
| IPI00021435 | 5701 | PSMC2 | 26S protease regulatory subunit _ | 5 | 0.99 | | | |
| IPI00022078 | 10397 | NDRG1 | N-myc downstream regulated gene 1 | 5 | 1.00 | | | |
| IPI00030920 | 29925 | GMPPB | GDP-mannose pyrophosphorylase B isoform 2 | 5 | 0.99 | | | |
| IPI00297982 | 1968 | EIF2S3 | Eukaryotic translation initiation factor 2 subunit 3 | 5 | 1.00 | | | |
| IPI00375704 | 5693 | PSMB5 | Proteasome beta 5 subunit | 5 | 1.00 | | | |
| IPI00020944 | 2222 | FDFT1 | Squalene synthetase | 4 | 1.00 | | | |
| IPI00021808 | 3035 | HARS | Histidyl-tRNA synthetase, cytoplasmic | 4 | 0.99 | | | |
| IPI00022228 | 3069 | HDLBP | High density lipoprotein binding protein | 4 | 0.99 | | | |
| IPI00026182 | 830 | CAPZA2 | F-actin capping protein alpha-2 subunit | 4 | 0.93 | | | |
| IPI00026530 | 3998 | LMAN1 | Lectin, mannose-binding, 1 | 4 | 1.00 | | | |

| | | | | | |
|---|---|---|---|---|---|
| IPI00028392 | 79098 | C1orf116 | Specifically androgen-regulated protein | 4 | 1.00 |
| IPI00145623 | 200916 | RPL22L1 | Similar to ribosomal protein L22 like 1 | 4 | 1.00 |
| IPI00215637 | 1654 | DDX3X | ATP-dependent RNA helicase DDX3X | 4 | 1.00 |
| IPI00216184 | 8301 | PICALM | Phosphatidylinositol-binding clathrin assembly protein | 4 | 1.00 |
| IPI00410341 | 79998 | ANKRD53 | Ankyrin repeat domain 53 | 4 | 0.91 |
| IPI00747748 | 5530 | PPP3CA | Protein phosphatase 3, catalytic subunit, alpha isoform | 4 | 0.98 |
| IPI00793665 | 5213 | PFKM | Phosphofructokinase, muscle | 4 | 0.99 |
| IPI00794973 | 327 | APEH | N-acylaminoacyl-peptide hydrolase | 4 | 0.99 |

MS = Mass spectra, P = Protein probability
