# Supplementary material for: Proteomic Interrogation of Androgen Action in Prostate Cancer Cells Reveals Roles of Aminoacyl tRNA Synthetases
Source: PLoS One. 2009 Sep 18;4(9):e7075. doi: 10.1371/journal.pone.0007075 (PMC2740864; doi:10.1371/journal.pone.0007075)
Supplement: Table S4 — (0.01 MB PDF) [file pone.0007075.s006.pdf]

Table S4 Proteins identified as androgen down-regulated through MudPIT LC-MS/MS

IPI_ID	Entrez_ID	Symbol	Protein Description	MS_ R1881	P-R1881	MS_ Control	P_ Control	Ratio (R1881/ Control)
IPI00215911	328	APEX1	DNA-(apurinic or apyrimidinic site) lyase	4	1.00	17	1.00	0.25
IPI00477179	9188	DDX21	Isoform 2 of Nucleolar RNA helicase 2	2	0.88	9	1.00	0.23
IPI00024403	8895	CPNE3	Copine-3	2	1.00	9	1.00	0.23
IPI00001734	29968	PSAT1	Isoform 1 of Phosphoserine aminotransferase	1	0.22	5	1.00	0.21
IPI00795088	7520	XRCC5	Hypothetical protein XRCC5	2	0.95	10	1.00	0.21
IPI00550308	10137	RBM12	RNA-binding protein 12	1	0.40	5	0.99	0.21
IPI00418497	92609	TIMM50	Import inner membrane translocase subunit TIM50	1	0.82	5	0.96	0.21
IPI00297396	57455	REXO1	RNA exonuclease 1 homolog	1	0.95	5	0.97	0.21
IPI00009032	6741	SSB	Lupus La protein	2	0.75	11	1.00	0.19
IPI00413611	7150	TOP1	DNA topoisomerase 1	2	0.96	11	1.00	0.19
IPI00385944	57003	CCDC47	Isoform 1 of Coiled-coil domain-containing protein 47 precursor	2	0.97	11	0.98	0.19
IPI00793839	1431	CS	Citrate synthase, mitochondrial precursor	2	0.30	11	1.00	0.19
IPI00398949	387841	LOC387841	Similar to ribosomal protein L13a	2	1.00	11	1.00	0.19
IPI00008433	6193	RPS5	40S ribosomal protein S5	2	0.84	12	1.00	0.18
IPI00010700	7916	BAT2	Isoform 1 of Large proline-rich protein BAT2	1	1.00	6	1.00	0.18
IPI00015920	1468	SLC25A10	Isoform 1 of Mitochondrial dicarboxylate carrier	1	0.83	6	1.00	0.18
IPI00021926	5706	PSMC6	26S protease regulatory subunit S10B	1	1.00	6	1.00	0.18
IPI00619903	56886	UGCGL1	UDP-glucose:glycoprotein glucosyltransferase 1 precursor	1	0.69	6	1.00	0.18
IPI00397509	55660	PRPF40A	Isoform 4 of Pre-mRNA-processing factor 40 homolog A	1	0.98	6	1.00	0.18
IPI00220219	9276	COPB2	Coatomer subunit beta	1	1.00	6	1.00	0.18
IPI00335132	3146	HMGB1	Similar to High mobility group protein 1	7	1.00	44	1.00	0.17
IPI00550247	11224	RPL35	Ribosomal protein L35	2	1.00	13	1.00	0.16
IPI00007074	8565	YARS	Tyrosyl-tRNA synthetetase, cytoplasmic	1	0.83	7	1.00	0.15
IPI00219005	2288	FKBP4	FK506-binding protein 4	1	0.43	7	1.00	0.15
IPI00219485	6625	SNRP70	Isoform 4 of U1 small nuclear ribonucleoprotein 70 kDa	1	0.98	7	1.00	0.15
IPI00643486	494115	RBMXL1	Novel protein similar to RNA binding motif protein, X-linked	1	0.90	7	0.99	0.15
IPI00018842	9324	HMGN3	High mobility group nucleosome-binding domain-containing protein 3	2	0.62	17	0.97	0.12

IPI00025039	2091	FBL	Fibrillarin	2	0.95	20	1.00	0.11
IPI00021785	1329	COX5B	Cytochrome c oxidase subunit 5B, mitochondrial precursor	1	1.00	11	1.00	0.10
IPI00745518	4134	MAP4	Microtubule-associated protein 4 isoform 1 variant (Fragment)	1	0.98	12	1.00	0.09
IPI00014186	463	ATBF1	Isoform A of Alpha-fetoprotein enhancer-binding protein	1	0.35	13	0.98	0.08
IPI00439415	1975	EIF4B	Eukaryotic translation initiation factor 4B			12	1.00	
IPI00554711	3728	JUP	Junction plakoglobin			8	1.00	
IPI00220484	10473	HMGN4	High-mobility group nucleosome-binding domain-containing protein 4			7	1.00	
IPI00011913	10949	HNRPA0	Heterogeneous nuclear ribonucleoprotein A0			6	1.00	
IPI00029264	1537	CYC1	Cytochrome c1, heme protein, mitochondrial precursor			6	1.00	
IPI00294159	6576	SLC25A1	Tricarboxylate transport protein, mitochondrial precursor			6	1.00	
IPI00554469	10989	IMMT	Isoform 2 of Mitochondrial inner membrane protein			6	1.00	
IPI00619966	1728	NQO1	NAD(P)H menadione oxidoreductase 1, dioxin-inducible isoform b			6	1.00	
IPI00642097	23708	GSPT2	Peptide chain release factor 3			6	1.00	
IPI00012074	10236	HNRPR	Heterogeneous nuclear ribonucleoprotein R			5	1.00	
IPI00012831	10542	HBXIP	hepatitis B virus x-interacting protein			5	1.00	
IPI00216654	9221	NOLC1	Isoform Beta of Nucleolar phosphoprotein p130			5	1.00	
IPI00645020	10465	PPIH	Peptidyl-prolyl cis-trans isomerase H			5	1.00	
IPI00749432	55740	ENAH	Isoform 1 of Protein enabled homolog			5	1.00	
IPI00027834	3191	HNRPL	heterogeneous nuclear ribonucleoprotein L isoform a			5	1.00	
IPI00003968	4704	NDUFA9	NADH dehydrogenase (ubiquinone) 1 alpha subcomplex, 9			4	0.97	
IPI00004669	2590	GALNT2	Polypeptide N-acetylgalactosaminyltransferase			4	0.99	
IPI00010346	57486	NLN	Neurolysin, mitochondrial precursor			4	0.97	
IPI00024387	3169	FOXA1	Hepatocyte nuclear factor 3-alpha			4	1.00	
IPI00032158	80155	NARG1	Isoform 2 of NMDA receptor-regulated protein 1			4	1.00	
IPI00220038	51593	ARS2	Isoform B of Arsenite-resistance protein 2			4	1.00	

IPI00479565	84193	SETD3	Isoform 1 of SET domain-containing protein 3	4	1.00
IPI00479997	3925	STMN1	Stathmin	4	0.87
IPI00644515	7019	TFAM	Transcription factor A, mitochondrial precursor	4	1.00
IPI00657954	9879	DDX46	Probable ATP-dependent RNA helicase DDX46	4	1.00
IPI00794543	808	CALM3	CALM3 protein	4	1.00

MS = Mass spectra, P = Protein probability
