# Supplementary material for: Proteomic Interrogation of Androgen Action in Prostate Cancer Cells Reveals Roles of Aminoacyl tRNA Synthetases
Source: PLoS One. 2009 Sep 18;4(9):e7075. doi: 10.1371/journal.pone.0007075 (PMC2740864; doi:10.1371/journal.pone.0007075)
Supplement: Table S5 — (0.01 MB PDF) [file pone.0007075.s007.pdf]

Table S5 Oligonucleotide primers used for ChIP-PCR

Gene	Accession	Primer name	Sequences
GARS	NM_002047	GARS_pF1	3'-ctggcccagaaagtactcca-5'
		GARS_pR1	3'-agattggaaggggaaggaag-5'
GAPDH	BC025925	GAPDH pF2	3'-tcgacagtcagccgcatct-5'
		GAPDH pR2	3'-ctagcctcccgggtttctct-5'
